# New species of *Indocloeon* Müller-Liebenau from South-East Asia (Ephemeroptera, Baetidae)

**DOI:** 10.3897/zookeys.723.20578

**Published:** 2017-12-18

**Authors:** Thomas Kaltenbach, Jean-Luc Gattolliat

**Affiliations:** 1 Museum of Zoology, Palais de Rumine, Place Riponne 6, CH-1005 Lausanne, Switzerland; 2 University of Lausanne (UNIL), Department of Ecology and Evolution, CH-1015 Lausanne, Switzerland; 3 Sonnenweg 1, CH-3280 Greng, Switzerland

**Keywords:** Brunei, COI, distribution, Indonesia, Oriental Region, taxonomy

## Abstract

One new species of *Indocloeon* Müller-Liebenau from Brunei, *I. spathasetis* **sp. n.**, and one new species from Indonesia, *I. timorense* **sp. n.**, are described and illustrated based on their larvae. The total number of known *Indocloeon* species increases from two to four and the generic attributes of the larvae are amended based on the examination of the new species. Results on the genetics of some species (COI) as well as comments on the distribution of *I. indonesiae* Kluge are also provided.

## Introduction

The genus *Indocloeon* was established by Müller-Liebenau (1982) for unusual larvae collected in Sri Lanka; the genus was originally monotypic including *Indocloeon primum* Müller-Liebenau. The genus was mainly characterised by a brush of setae between prostheca and mola of the right mandible, a slender maxillary palp reaching far beyond galea-lacinia, a labial palp with a pointed distomedial protuberance, slender legs with nearly parallel margins, the outer margin of the femur with strong short setae, the outer margin of the tibia with only two apical setae and no setae at outer margin of the tarsus, moderately elongate, pointed claws with two rows of denticles and the posterior margin of terga with long spines, which are fused at base and pointed at apex (Müller-Liebenau 1982). It belongs to the plesiomorphon Protopatellata lineage within the family Baetidae, as the patella-tibial suture is only developed on middle and hind legs of all stages (Kluge 2012). A second species was described by Kluge (2012) from Indonesia, *Indocloeon indonesiae* Kluge. He also provided a complete re-description of the genus and of *I. primum* as well as the first description of the imaginal stages. Here we describe two new species of *Indocloeon* based on material recently collected in Brunei Darussalam and Indonesia. Further we investigated the genetics of three species (cytochrome oxidase subunit 1, COI). All species of *Indocloeon* are morphologically and genetically well differentiated. The discovery of these new species implied the revision of part of the generic characters.

## Materials and methods

The specimens were collected by Kate Baker (King’s College London, UK) in the Ulu Temburong National Park in Brunei Darussalam with surber and kick-sampling methods (Baker et al. 2016a, b, 2017a, b) and by Michael Balke (Zoologische Staatssammlung München, ZSM, Germany) on the islands of Flores, Sumbawa, and Timor (Indonesia).

The specimens were preserved in 70%–96% ethanol. The dissection of larvae was done in Cellosolve (2-Ethoxyethanol) with subsequent mounting on slides with Euparal liquid, using an Olympus SZX7 stereomicroscope.

The DNA of part of the specimens was extracted using non-destructive methods allowing subsequent morphological analysis (see Vuataz et al. 2011 for details). We amplified a 658 bp fragment of the mitochondrial gene cytochrome oxidase subunit 1 (COI) using the primers LCO 1490 (GGTCAACAAATCATAAAGATATTGG) and HCO 2198 (TAAACTTCAGGGTGACCAAAAAATCA) (Folmer et al. 1994). The polymerase chain reaction was conducted with an initial denaturation temperature of 98 °C for 30 sec followed by a total of 37 cycles with denaturation temperature of 98 °C for 10 sec, an annealing temperature of 50 °C for 30 sec and an extension at 72 °C for 30 sec, final extension at 72 °C for 2 min. Sequencing was done with the method of Sanger (Sanger et al. 1977). The genetic variability between specimens was estimated using the Kimura-2-parameter distances (Kimura 1980) calculated with the program MEGA 7 (Kumar et al. 2016, http://www.megasoftware.net). The GenBank accession numbers are given in Table 1.

**Table 1.** Sequenced specimens.

Species	Locality	Specimen catalog #	GenBank # (COI)	GenSeq Nomenclature
*I. spathasetis* sp. n.	Brunei	GBIFCH 00280816	MF414701	genseq-1 COI
*I. spathasetis* sp. n.	Brunei	GBIFCH 00280817	MF414702	genseq-2 COI
*I. indonesiae*	Flores	GBIFCH 00280818	MF414703	genseq-4 COI
*I. indonesiae*	Flores	GBIFCH 00280819	MF414704	genseq-4 COI
*I. indonesiae*	Sumbawa	GBIFCH 00280820	MF414705	genseq-4 COI
*I. indonesiae*	Sumbawa	GBIFCH 00280821	MF414706	genseq-4 COI
*I. timorense* sp. n.	Timor	GBIFCH 00280822	MF414707	genseq-1 COI

Drawings were made using an Olympus BX43 microscope. Photographs of larvae were taken using a Canon EOS 6D camera and the Visionary Digital Passport imaging system (http://www.duninc.com) and processed with the programs Adobe PhotoShop Lightroom (http://www.adobe.com) and Helicon Focus version 5.3 (http://www.heliconsoft.com). Photographs were subsequently enhanced with Adobe Photoshop Elements 13.

The distribution map was generated with the program Simple Mapper (http://research.amnh.org/pbi/maps) and the program GEOLocate (http://www.museum.tulane.edu/geolocate/web/WebGeoref.aspx) was used to attribute approximate GPS coordinates to sample locations of Müller-Liebenau (1982) and Kluge (2012).

The taxonomic descriptions presented herein were generated with a DELTA (Dallwitz 1980, Dallwitz et al. 1999) database containing the morphological states of characters of the new *Indocloeon* species.

## Results

### New species descriptions

#### Indocloeon spathasetis sp. n.

Taxon classificationAnimaliaORDOFAMILIA

http://zoobank.org/29C62FD3-7205-4ADB-B658-6740648D746B

**Figure 1.**
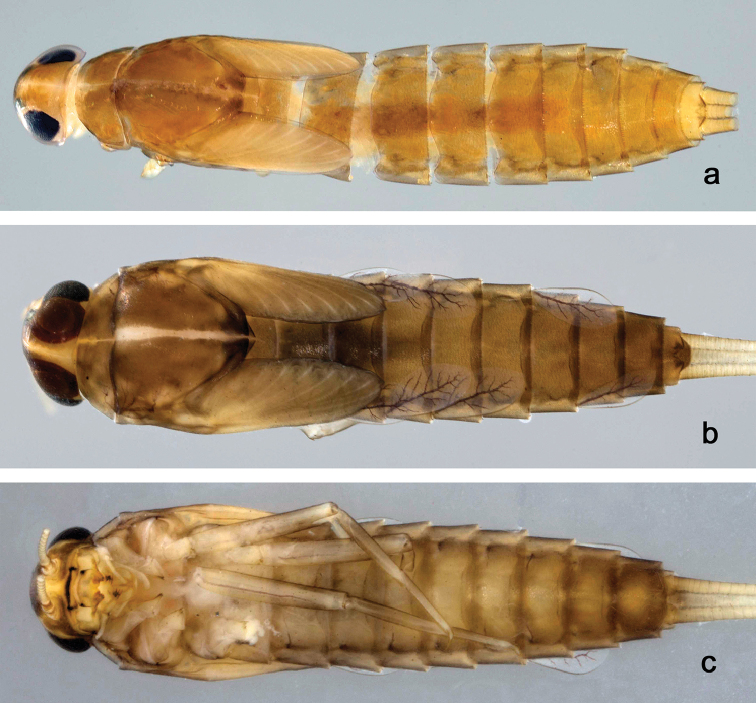
Habitus, larvae: **a** *Indocloeon spathasetis* sp. n., dorsal view **b** *Indocloeon timorense* sp. n., dorsal view **c** *Indocloeon timorense* sp. n., ventral view.

**Figure 2.**
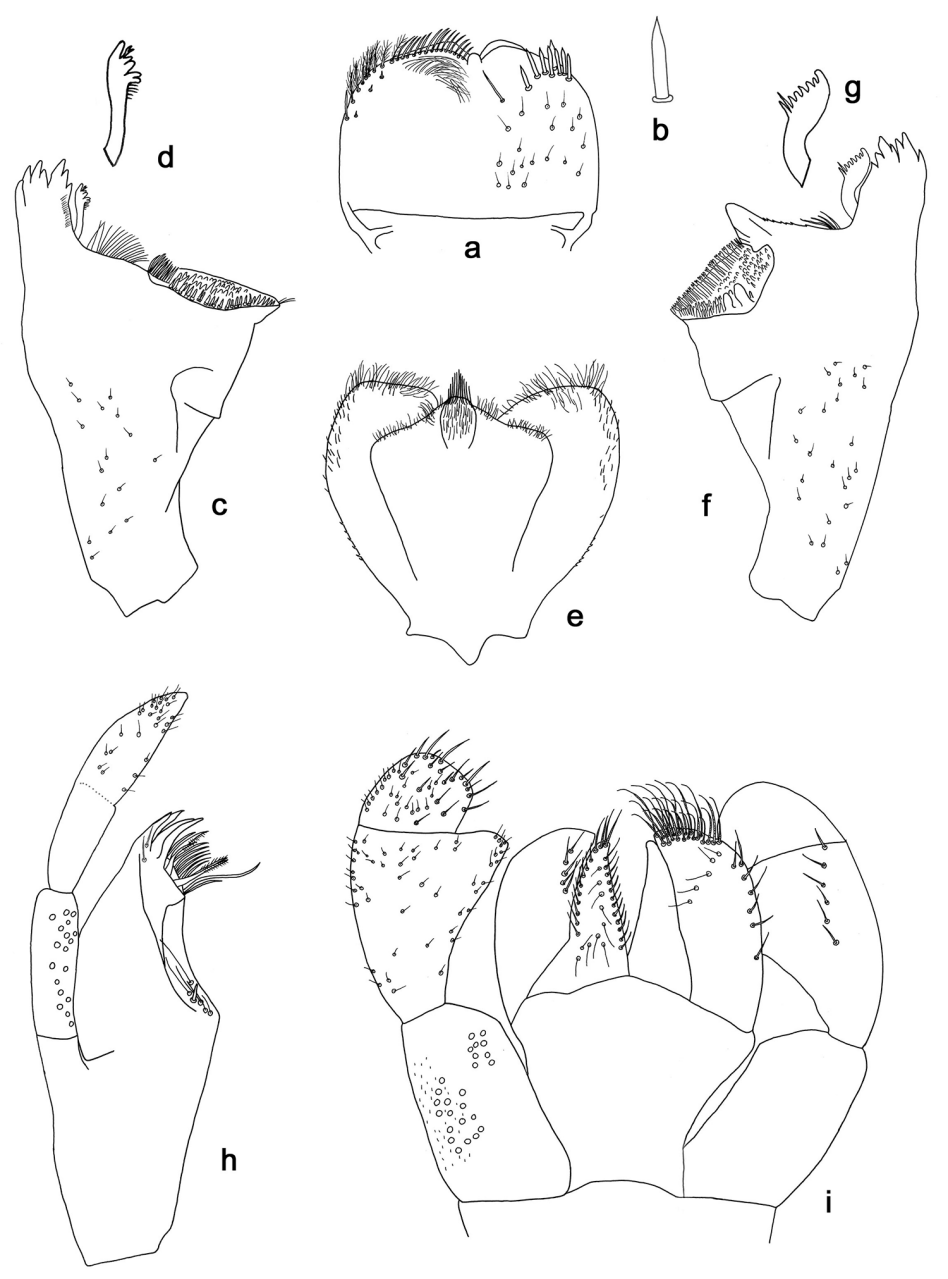
*Indocloeon spathasetis* sp. n., larva morphology: **a** Labrum **b** Seta of the labrum dorsal submarginal arc **c** Right mandible **d** Right prostheca **e** Hypopharynx **f** Left mandible **g** Left prostheca **h** Maxilla **i** Labium.

**Figure 3.**
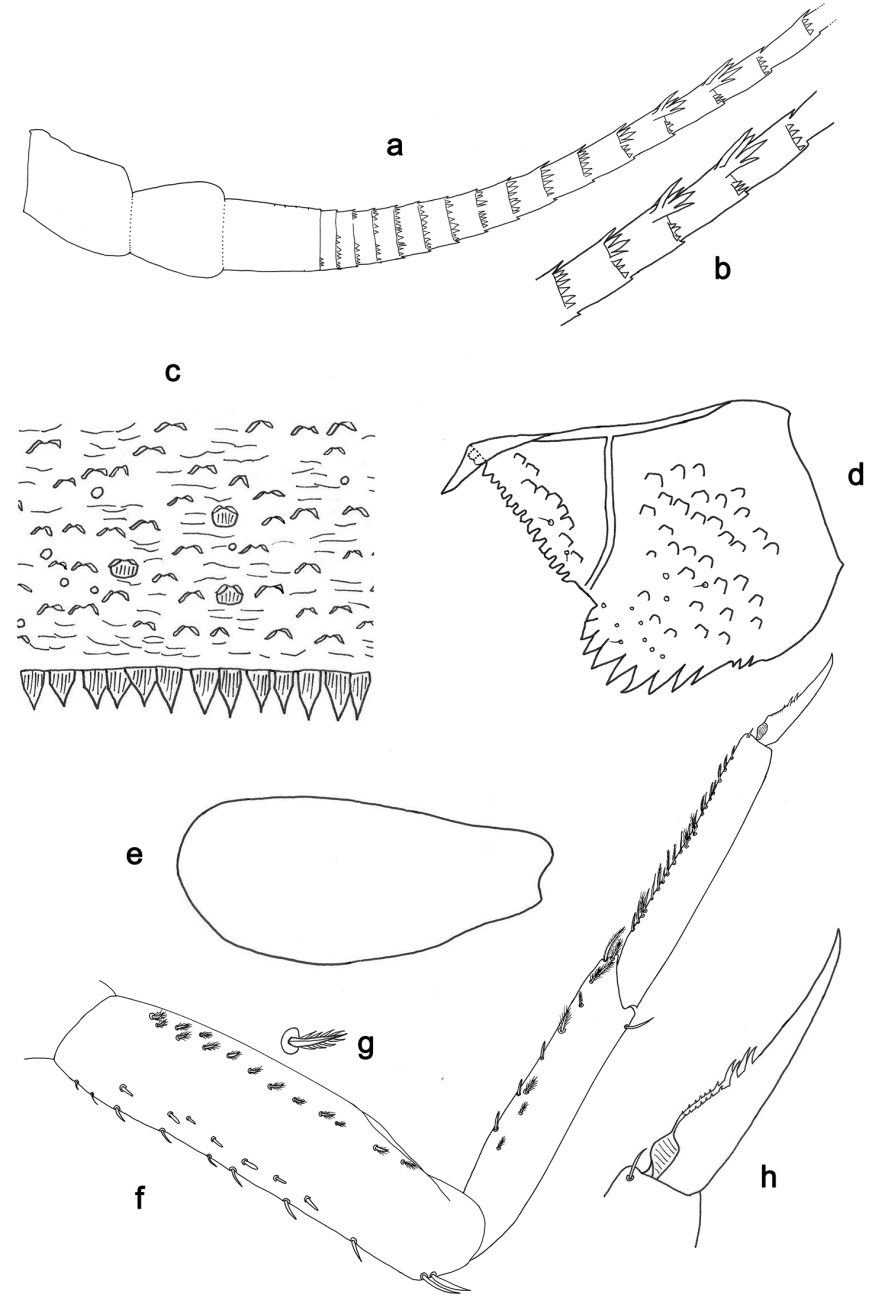
*Indocloeon spathasetis* sp. n., larva morphology: **a** Left antenna, proximal part **b** Left antenna, detail of middle segments **c** Tergum IV **d** Paraproct **e** Gill VI **f** Foreleg **g** Foreleg, bipectinate seta **h** Fore tarsal claw.

Figures 1a , 2 , 3 , 6a–c

##### Diagnosis.

**Larva.** Following combination of characters: A) middle length of antenna with conspicuous large spines at outer lateral margin (Fig. 3a, b); B) labrum with submarginal dorsal arc of setae composed of a medium apically pointed seta plus eight medium, clearly spatulate and apically pointed setae (Fig. 2a, b); C) distomedial protuberance at segment II of labial palp well developed, triangular, apically slightly rounded with partly flattened margin (Fig. 2i); D) claw with two rows of denticles, each with two larger denticles apically and many small denticles basally (Fig. 3h).

##### Description.

**Larva** (Figs 1a, 2, 3). Body length 3.7 mm; antenna: approximately twice as long as head length.

*Colouration*. Head, thorax and abdomen dorsally brown (Fig. 1a). Head and thorax dorsally with bright longitudinal line, forewing pads with bright striation, tergum X light brown. Thorax and abdomen ventrally brown. Legs colourless with a distomedial brown area on femur, medial on tibia and proximal on tarsus. Caudal filaments light brown.

*Antenna* (Fig. 3a, b) with scape and pedicel subcylindrical; flagellum with long spines on apex of each segment and with scales. Middle part of flagellum with very large and long spines on apex of a few segments at outer lateral margin.

*Labrum* (Fig. 2a). Rectangular, length 0.7× maximum width. Distal margin with medial emargination and small process. Dorsally with medium, fine, simple setae scattered over surface; submarginal dorsal arc of setae composed of one medium, apically pointed central seta plus eight medium, spatulate, apically pointed setae. Ventrally with submarginal row of setae composed of lateral and partly anterolateral feathery setae and medial as well as partly anterolateral simple setae; short, spine-like setae near lateral and anterolateral margin.

*Right mandible* (Fig. 2c, d). Incisors fused. Outer and inner sets of denticles with 4 + 3 denticles respectively. Inner margin of innermost denticle with a row of thin setae. Prostheca robust, apically denticulate (Fig. 2d). Margin between prostheca and mola straight, tuft of setae present. Tuft of spine-like setae at base of mola absent. Tuft of setae at apex of mola present.

*Left mandible* (Fig. 2f, g). Incisors fused. Outer and inner sets of denticles with 3 + 4 denticles respectively. Prostheca robust, apically denticulate, with comb-shape structure (Fig. 2g). Margin between prostheca and mola straight, with minute denticles towards subtriangular process. Tuft of setae between prostheca and mola small and directed proximally. Tuft of spine-like setae at base of mola absent. Subtriangular process wide, slightly above level of area between prostheca and mola. Denticles of mola apically constricted. Tuft of setae at apex of mola absent.

Both *mandibles* with lateral margins almost straight. Basal half with fine, simple setae scattered over dorsal surface.

*Hypopharynx* (Fig. 2e). Lingua shorter than superlingua. Lingua longer than broad. Medial tuft of setae present; distal half laterally expanded. Superlingua apical margin rounded; lateral margin rounded; simple setae scattered over lateral and distal margin, finer and longer at distal margin.

*Maxilla* (Fig. 2h). With two simple, robust apical setae under crown. Inner dorsal row of setae with three denti-setae, distal denti-seta teeth-like, middle and proximal denti-setae slender and pectinate. Medially with one spine-like seta and five long, simple setae. Maxillary palp 1.5× as long as length of galea-lacinia; three segmented, segment II and III nearly fused; setae on maxillary palp fine, simple, scattered over surface of segment III, especially at apex. Palp segment II 0.7× length of segment I. Palp segment III 1.3× length of segment II. Apex of last segment conical.

*Labium* (Fig. 2i). Glossa basally broad, narrowing toward apex, slightly shorter than paraglossa. Inner margin with 12 long, simple setae. Apex with four long, robust, pectinate setae. Outer margin with nine long, simple setae. Ventral surface with long, fine, simple, scattered setae. Paraglossa sub-rectangular, apex obliquely truncate and slightly rounded. Apex with three rows of robust, pectinate setae. Outer margin with row of seven long, spine-like setae. Dorsally with row of five long, simple setae. Ventrally with five long, simple setae near inner margin. Labial palp with segment I 0.7× length of segments II and III combined. Segment I covered with micropores and with tiny, robust, simple setae along outer margin. Segment II with triangular, apically slightly rounded distomedial protuberance; distomedial protuberance 0.5× width of base of segment III; inner margin with short, fine, simple setae, more abundant at apex; outer margin with short, fine, simple setae; dorsally with row of six long, simple setae. Segment III subquadrangular, asymmetrical; length 0.8× width; covered with long, simple setae and short, fine, simple setae, apically more dense.

*Hind wing pads* absent.

*Foreleg* (Fig. 3f, g, h). Ratio of foreleg 1.5:1.0:1.0:0.4. *Fore femur*. Length approximately 4× maximum width. Dorsally with row of eight curved, spine-like, short setae on margin and a row of seven stout and somewhat spatulate setae near margin; length of setae 0.2× maximum width of femur. Apex rounded; with two long, curved, spine-like setae. Ventrally with about 14 bipectinate, stout setae, predominantly arranged in one row. *Tibia*. Dorsally bare except one long, spine-like, curved seta near apex. Ventrally with bipectinate, stout setae on margin and close to margin, apical setae very long (pectination in lateral view difficult to see). Tibio-patellar suture absent. *Tarsus.* Dorsally bare. Ventrally with many bipectinate, stout setae on margin and close to margin (pectination in lateral view difficult to see). Tarsal claw with two rows of many minute denticles and two large apical denticles; subapical setae absent.

*Terga* (Fig. 3c). Surface with abundant scales or scale-bases and micropores. Posterior margin with row of irregular triangular or pentagonal spines.

*Gills* (Fig. 3e). On segments I – VII. Margin smooth. Tracheae restricted to main trunk. Gill I as long as length of segment II; lanceolate. Gill VII about 2/3 length of segment VIII; oblong.

*Paraproct* (Fig. 3d). With 12 marginal stout spines, laterally smaller. Surface with scale bases, micropores and a few short, fine, simple setae. Postero-lateral extension (cercotractor) with small marginal spines.

##### Etymology.

Refers to the noticeable spatulate submarginal dorsal setae of the labrum.

##### Distribution.

Only known from Brunei, but presence in other regions of Borneo such as Sarawak and Sabah (Malaysia) is possible as their fauna remains poorly known.

##### Biological aspects.

The specimens were collected in lowland tropical rainforest at an altitude of about 100 m a.s.l., directly at the confluence of small tributaries with large rivers (Belalong, Temburong) as well as in upstream pools of these tributaries (Fig. 6a, b, c). Substrates were predominantly characterised by cobbles and gravel.

##### Type-material.

**Holotype.** Larva (on slide, GBIFCH 00280816), Brunei, Temburong District, Ulu Temburong National Park, 4°32.77'N, 115°09.52'E, May 2014, leg. Kate Baker. **Paratypes.** Brunei, Temburong District, Ulu Temburong National Park, May 2014, leg. Kate Baker: larva (on slide, GBIFCH 00280817), 4°32.77'N, 115°09.52'E; two larvae (one on slide, GBIFCH 00465131; one in alcohol, GBIFCH 00515214), 4°33.67'N, 115°08.87'E; two larvae (one on slide, GBIFCH 00465130; one in alcohol, GBIFCH 00515213), 4°33.64'N, 115°09.07'E; larva (on slide, GBIFCH 00465132), 4°33.39'N, 115°10.03'E; larva (on slide, GBIFCH 00465133), 4°33.21'N, 115°09.31'E; two larvae (one on slide, GBIFCH 00465134; one in alcohol, GBIFCH 00515215), 4°32.87'N, 115°09.47'E. All material deposited in the Museum of Zoology Lausanne (MZL).

##### Additional material.

Five larvae (in alcohol, GBIFCH 00515216). Brunei, Temburong District, Ulu Temburong National Park, near Kuala Belalong Field Studies Centre, tributary to Temburong river, 4°33.21'N, 115°09.31'E, May 2014, leg. Kate Baker. Deposited in the Museum of Zoology Lausanne (MZL).

#### Indocloeon timorense sp. n.

Taxon classificationAnimaliaORDOFAMILIA

http://zoobank.org/DDD47504-FDE1-4FD1-BA0C-12C4DDA3DAF3

**Figure 4.**
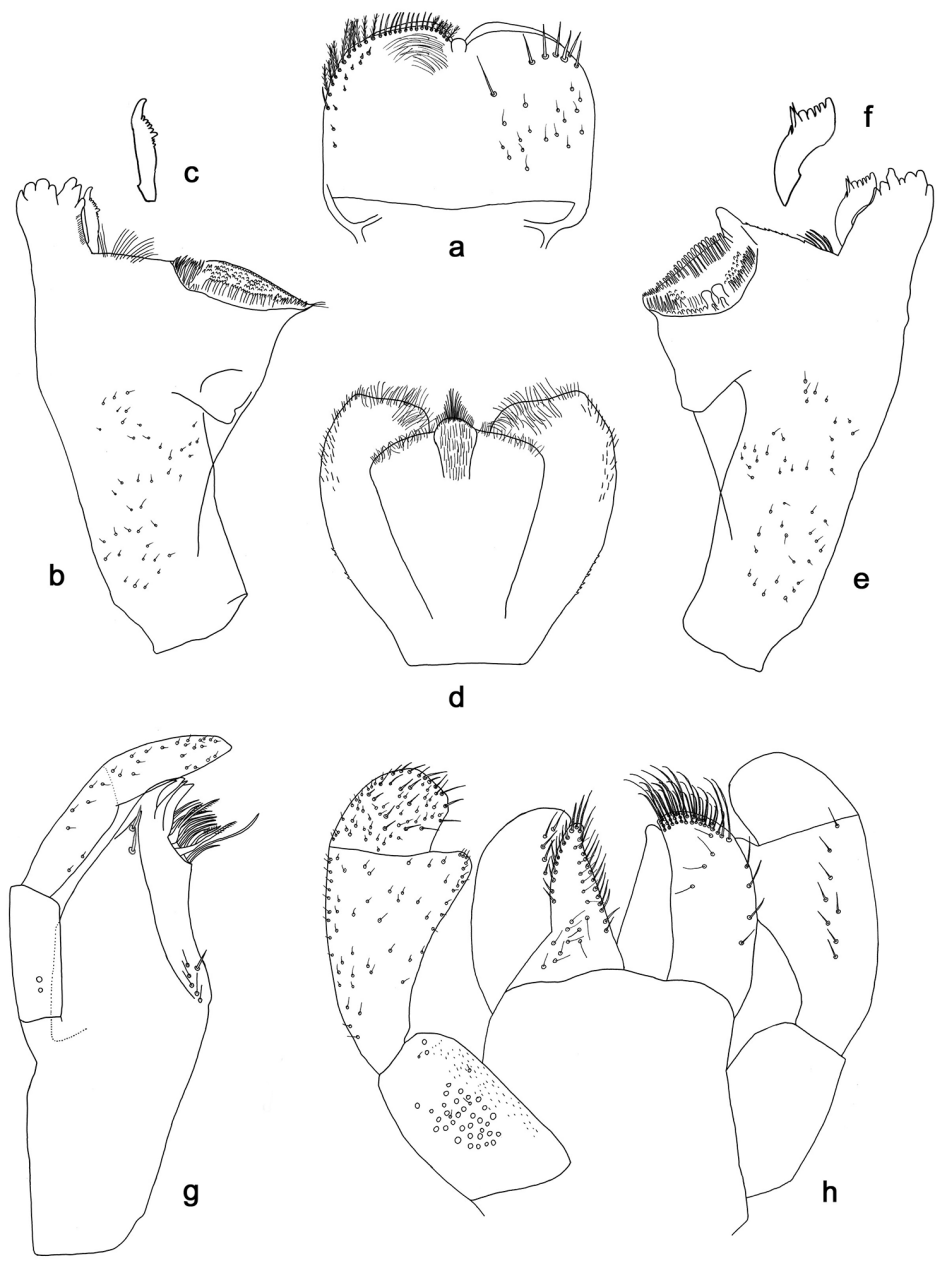
*Indocloeon timorense* sp. n., larva morphology: **a** Labrum **b** Right mandible **c** Right prostheca **d** Hypopharynx **e** Left mandible **f** Left prostheca **g** Maxilla **h** Labium.

**Figure 5.**
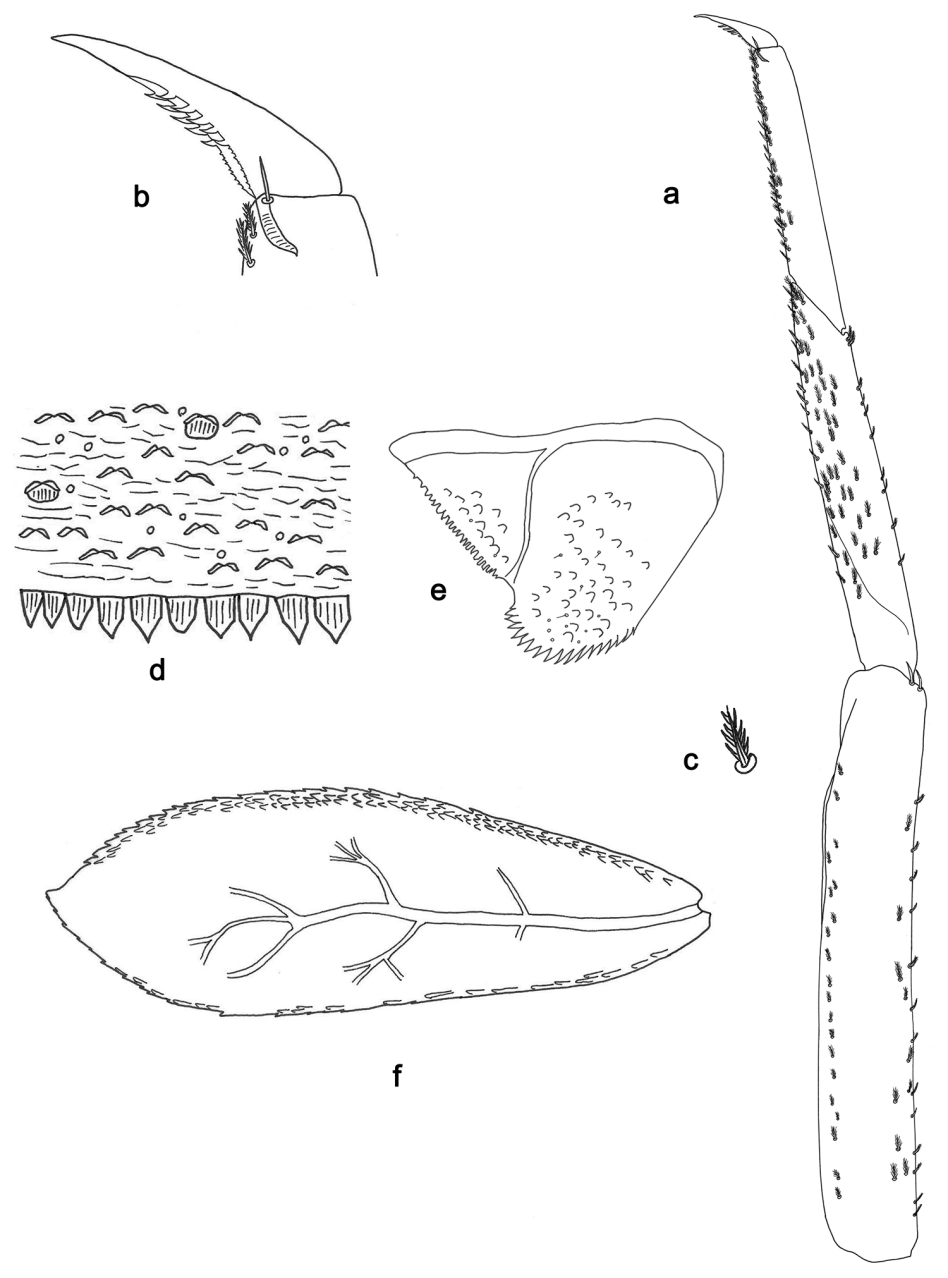
*Indocloeon timorense* sp. n., larva morphology: **a** Middle leg **b** Middle tarsal claw **c** Middle leg, bipectinate seta **d** Tergum IV **e** Paraproct **f** Gill VII.

Figures 1b, c , 4 , 5

##### Diagnosis.

**Larva.** Following combination of characters: A) labrum with submarginal arc of setae composed of one central medium, simple seta plus six medium, simple setae (Fig. 4a); B) distomedial protuberance at segment II of labial palp moderately developed, apically rounded (Fig. 4h); C) claw with two rows of denticles, each with five larger denticles apically and many small denticles basally (Fig. 5b); D) gills with serrate margin and pointed scales along margin (Fig. 5f).

##### Description.

**Larva** (Figs 1b, c, 4, 5). Body length 4.6 mm.

*Colouration*. Head, thorax and abdomen dorsally brown. Head and thorax with bright dorsal line, forewing pads with bright striation (Fig. 1b). Thorax and abdomen ventrally brown, legs brown (Fig. 1c). Caudal filaments colourless, but light brown at base.

*Antenna* with scape and pedicel subcylindrical; flagellum with broad spines on apex of each segment and with scales. In the middle part of the flagellum without large spines on apex of segments at outer lateral margin.

*Labrum* (Fig. 4a). Rectangular, length 0.7× maximum width. Distal margin with medial emargination and small process. Dorsally with medium, fine, simple setae scattered over surface; submarginal dorsal arc of setae composed of 1 + 6 long, apically pointed simple setae. Ventrally with submarginal row of setae composed of lateral long and medial shorter, feathery setae and anterolateral simple setae; ventral surface with short, spine-like setae near lateral and anterolateral margin.

*Right mandible* (Fig. 4b, c). Incisors fused. Outer and inner sets of denticles with 4 + 3 denticles respectively. Inner margin of innermost denticle with a row of thin setae. Prostheca robust, apicolaterally denticulate. Margin between prostheca and mola straight, tuft of setae present. Tuft of spine-like setae at base of mola absent. Tuft of setae at apex of mola present.

*Left mandible* (Fig. 4e, f). Incisors fused. Outer and inner sets of denticles with 4 + 3 denticles respectively. Prostheca robust, apically denticulate, with comb-shape structure. Margin between prostheca and mola straight, with minute denticles towards subtriangular process. Tuft of setae between prostheca and mola small and directed proximally. Tuft of spine-like setae at base of mola absent. Subtriangular process wide, slightly above level of area between prostheca and mola. Denticles of mola apically constricted. Tuft of setae at apex of mola absent.

Both *mandibles* with lateral margins almost straight. Basal half with fine, simple setae scattered over dorsal surface.

*Hypopharynx* (Fig. 4d). Lingua shorter than superlingua. Lingua longer than broad. Medial tuft of setae present; distal half laterally expanded. Superlingua apical margin slightly concave; lateral margin rounded; simple setae scattered over lateral and distal margin, finer and longer at distal margin.

*Maxilla* (Fig. 4g). With two simple, robust apical setae under crown. Inner dorsal row of setae with three denti-setae, distal denti-seta teeth-like, middle and proximal denti-setae slender and pectinate. Medially with one spine-like seta and five long, simple setae. Maxillary palp longer than length of galea-lacinia; three segmented; setae on maxillary palp fine, simple, scattered over surface of segments II and III. Palp segment II about as long as segment I. Palp segment III about as long as segment II. Apex segment III slightly pointed.

*Labium* (Fig. 4h). Glossa basally broad, narrowing toward apex. Slightly shorter than paraglossa. Inner margin with 13 long, simple setae. Apex with three long, robust, pectinate setae. Outer margin with nine long, simple setae. Ventral surface with long, fine, simple, scattered setae. Paraglossa sub-rectangular, apex obliquely truncate and slightly rounded. Apex with three rows of robust, pectinate setae. Outer margin with row of four long, spine-like setae. Dorsally with row of four long, simple setae. Ventrally with five long, simple setae near inner margin. Labial palp with segment I 0.6× length of segments II and III combined. Segment I covered with micropores and some short, fine, simple setae as well as with very short, robust, simple setae along inner margin. Segment II with apically rounded distomedial protuberance; distomedial protuberance 0.3× width of base of segment III; inner margin with short, fine, simple setae; outer margin with short, fine, simple setae; dorsally with row of eight long, simple setae. Segment III subquadrangular, asymmetrical; length subequal to width; covered with medium, simple setae and short, fine, simple setae.

*Hind wing pads* absent.

*Middle leg* (Fig. 5a, b, c). Ratio of middle leg 1.6:1.0:0.7:0.2. *Middle femur*. Length approximately 6× maximum width. Dorsally with 20–25 bipectinate, acute setae on margin and close to margin (pectination in lateral view difficult to see); length of setae 0.1× maximum width of femur. Apex rounded; with two long, curved, spine-like setae. Ventrally with around 20 bipectinate, stout setae, predominantly arranged in one row. *Tibia*. Dorsally with row of ten bipectinate, stout setae on margin, two of them near apex (pectination in lateral view difficult to see). Ventrally with many bipectinate, stout setae on margin and close to margin (pectination in lateral view difficult to see). Anterior surface with many bipectinate, stout setae. Tibio-patellar suture present on basal half. *Tarsus.* Dorsally bare. Ventrally with many bipectinate, stout setae on margin and close to margin (pectination in lateral view difficult to see). Tarsal claw with two rows of numerous minute denticles and five large, apical denticles; subapical setae absent.

*Terga* (Fig. 5d). Surface with abundant scales or scale-bases and micropores. Posterior margin with row of irregular triangular or pentagonal spines.

*Gills* (Fig. 5f). On segments I – VII. Margin serrate with small spines and with pointed scales along margin. Tracheae extending from main trunk to inner and outer margins. Gill I little longer than segment II; oblong. Gill IV as long as length of segments V and VI combined; oval. Gill VII as long as length of segments VIII and IX; oblong.

*Paraproct* (Fig. 5e). With 18 marginal stout spines, laterally smaller. Surface with scale bases, micropores, and a few short, fine, simple setae. Postero-lateral extension (cercotractor) with small marginal spines.

##### Etymology.

After the type locality, the island of Timor (Indonesia).

##### Distribution.

Indonesia: Timor.

##### Biological aspects.

The specimen was collected at an altitude of 1580 m a.s.l.

##### Type-material.

**Holotype.** Larva (on slide, GBIFCH 00280822), Indonesia, Timor, Mt. Mutis, 1580 m, 01.10.2011, 9°38.12'S, 124°12.80'E, leg. M. Balke. Temporary deposited in the Museum of Zoology Lausanne (MZL) before definitely housed in the Museum Zoologicum Bogoriense (MZB) in Indonesia.

### Distribution

In addition to the two new species found in Brunei and Timor, the occurrence of *Indocloeon indonesiae* on two further Indonesian islands (Flores and Sumbawa) is documented, additionally to Lombok, from where it was described by Kluge (2012). Thus the genus *Indocloeon* is presently known from partly distant locations in the Oriental realm, Sri Lanka on one hand and several islands of Indonesia as well as Brunei on the other (Fig. 7). The GPS coordinates of the new locations are given in Table 2.

**Figure 6.**
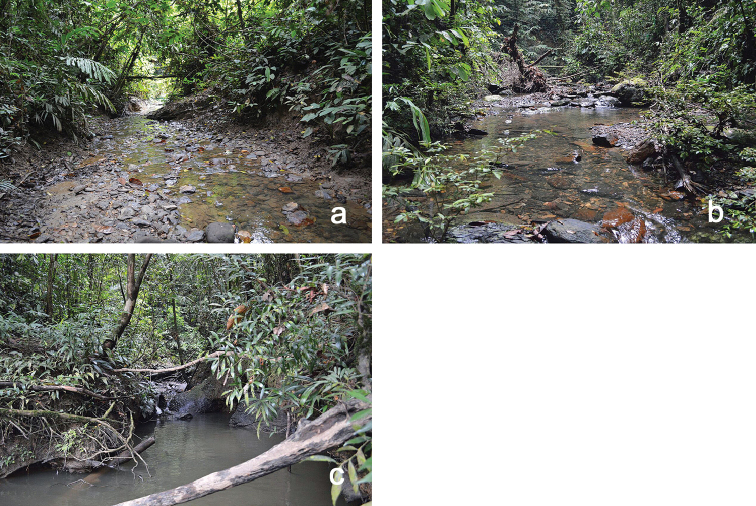
General aspects of *Indocloeon* larval habitats: **a, b** *I. spathasetis* sp. n., tributaries upstream **c** *I. spathasetis* sp. n., tributary confluence with main river. Photographs by Kate Baker (King’s College London).

**Figure 7.**
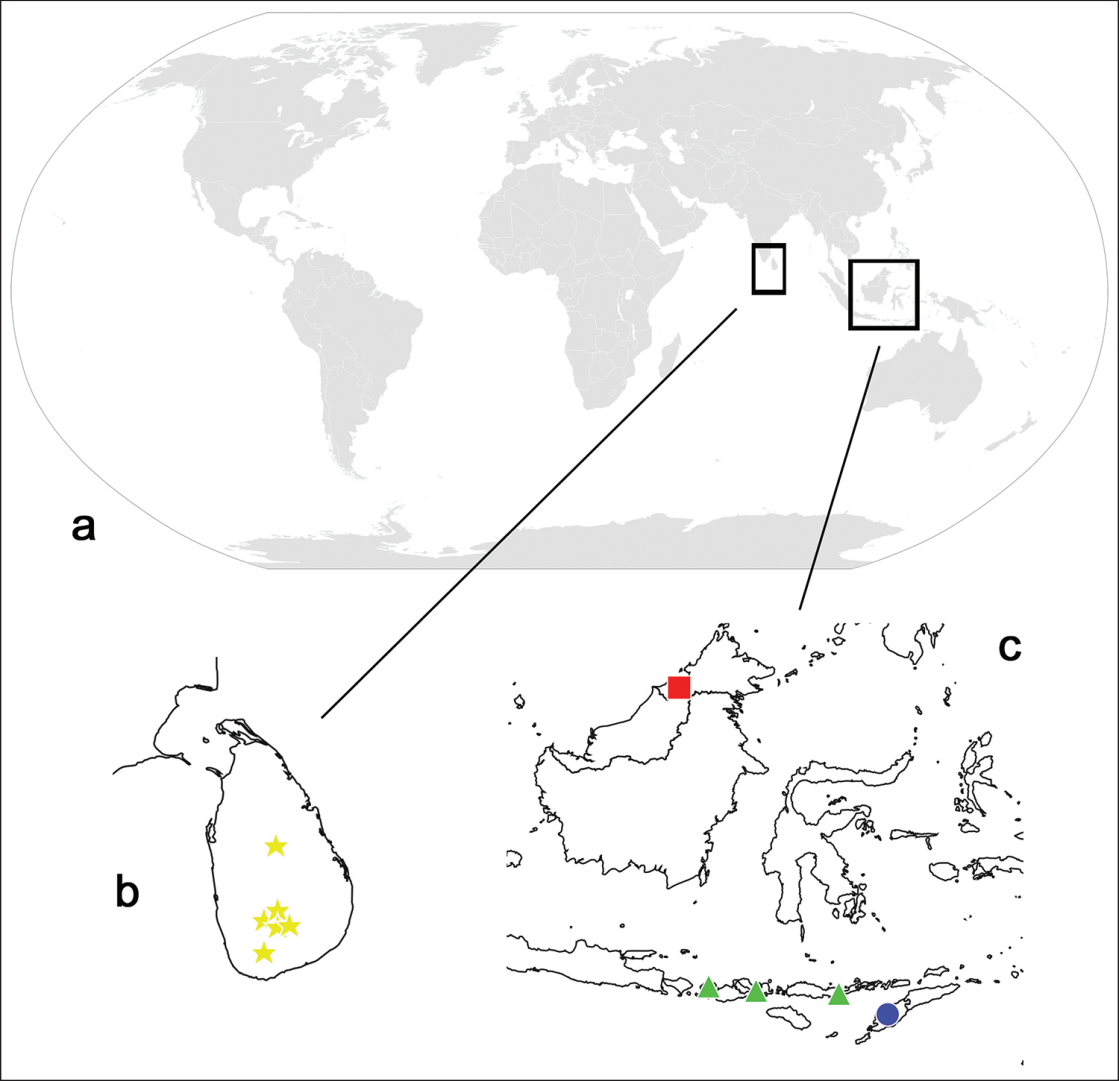
Distribution of *Indocloeon*: **a** World map (http://www.pixabay.com) **b** Sri Lanka **c** Indonesia (partim) and Brunei (yellow stars, *I. primum*; red square, *I. spathasetis* sp. n.; green triangles, *I. indonesiae* (from left to right: islands of Lombok, Sumbawa, Flores); blue spot, *I. timorense* sp. n. (island Timor)).

**Table 2.** GPS coordinates of locations of examined specimens.

**Species**	**Locality**	**GPS coordinates**
*I. indonesiae*	Sumbawa	8°38.54'S, 118°30.31'E
	Flores	8°42.92'S, 122°04.41'E
*I. timorense* sp. n.	Timor	9°38.12'S, 124°12.80'E
*I. spathasetis* sp. n.	Brunei	4°32.77'N, 115°09.52'E
		4°33.64'N, 115°09.07'E
		4°33.67'N, 115°08.87'E
		4°33.21'N, 115°09.31'E
		4°33.39'N, 115°10.03'E
		4°32.87'N, 115°09.47'E

### Genetics

COI sequences were obtained from two specimens of *I. indonesiae* from Flores (Indonesia) and two from Sumbawa (Indonesia), one specimen of *I. timorense* sp. n. from Timor (Indonesia) and two specimens of *I. spathasetis* sp. n. from Brunei. Only very limited genetic distances were found between specimens of *I. indonesiae* from Flores and Sumbawa (Table 3). The genetic distances between the three species are much higher than 3.5%, generally considered as a likely maximal value for intraspecific divergence (Hebert et al. 2003, Ball et al. 2005, Zhou et al. 2010), *I. timorense* sp. n. being apparently closer to *I. indonesiae* than *I. spathasetis* sp. n. (Table 3).

**Table 3.** Genetic distances (COI) between some *Indocloeon* species, using the Kimura 2-parameter.

	*I. indonesiae*	*I. indonesiae*	*I. timorense* sp.n.
	Flores	Sumbawa	Timor
*I. indonesiae*	0.01		
Sumbawa			
*I. timorense* sp. n.	0.15	0.14	
Timor			
*I. spathasetis* sp. n.	0.20	0.20	0.22
Brunei			

## Discussion

The generic description was given by Müller-Liebenau (1982) and later more detailed by Kluge (2012). The new species are attributed to *Indocloeon* based on the following characters: frons between antennae forms an elevation more or less narrowing anteriorly; cuticle of abdominal terga nearly unicolour; left mandible with incisors fused, distalmost denticle turned ventrally and terminates before apex, prostheca massive, with a tuft of setae between prostheca and mola; right mandible with incisors fused, distalmost denticle turned ventrally and terminates before apex, prostheca stick-like, with a row of setae between prostheca and mola; hypopharynx with a median tuft of stout setae; legs slender, femora slender and nearly parallel-sided; femora, tibiae and tarsi have stout, pointed, bipectinate setae; outer margin of femur lacks longitudinal row of stout setae, but irregular pectinate setae can be confused with such row (*I. timorense* sp. n. only; *I. spathasetis* sp. n. does have a longitudinal row of stout setae at the dorsal margin of the femur); claw slender, slightly bent, with two rows of denticles; terga with fine, longitudinally striated scales, situated in angulate nests.

Based on the additional two new species, the following adaptations to the generic diagnosis are proposed: the maxillary palp differs between the species, always with three segments and longer than galea-lacinia, but may be very long and slender (*I. primum*, fig. 1e in Müller-Liebenau 1982; *I. indonesiae*, fig. 5 in Kluge 2012) or less long and stouter (*I. spathasetis* sp. n., fig. 2h). The claw is slender, slightly bent with two rows of denticles; some of the distalmost denticles are considerably larger and directed distally, the other ones are very small. *Indocloeon primum*, *I. indonesiae* and *I. timorense* sp. n. possess bipectinate setae along the dorsal margin of the femur. In contrary we found two rows of simple or slightly spatulate setae along the dorsal margin of the femur in *I. spathasetis* sp. n. from Brunei and no pectinate structure can be observed with an optical microscope with a magnification of up to 600 times.

Taking into account the taxa described herein, the genus *Indocloeon* now encompasses four species, all of them occurring in the Oriental realm. In general, the different known species of *Indocloeon* can be easily identified by a combination of a few characters and often even by a single unique character. Overall important characters are the dorsal submarginal arc of setae of the labrum, the shape of the distomedial protuberance of segment II of the labial palp, the shape of the maxillary palp, the number of larger denticles of the claw and also the number of marginal spines of the paraproct.

*Indocloeon primum* can be recognised by the pointed distomedial protuberance of segment II of the labial palp, the long and slender maxillary palp with a very short segment III and the basally fused spines at the posterior margin of the terga (Müller-Liebenau 1982); *I. indonesiae* by the long and slender maxillary palp with three long segments (Kluge 2012) and *I. spathasetis* sp. n. by the distinctly spatulate, acute dorsal submarginal arc setae on the labrum and the long spines on the apex of a few segments at outer lateral margin in the middle part of the flagellum.

Contrary to mouthparts, legs and abdomen, which are showing important specific differences within *Indocloeon*, both mandibles seem to be quite uniform inside the genus.

*Indocloeon indonesiae* and *I. timorense* sp. n. are morphologically the most similar species, especially when referring to the labrum submarginal arc of setae and the labial palps. However, the maxillary palps are clearly different. This similarity is corroborated by the genetic distance (K2P) based on COI (Table 3). Both species seem to be closely related to each other, but their status as different species is confirmed by their K2P distance of 0.14 and 0.15 respectively, which is clearly of the interspecific range (Ball et al. 2005).

From *I. indonesiae* COI sequences were obtained from specimens of the Indonesian islands Sumbawa and Flores, but not from Lombok from where the species was originally described (Kluge 2012). There are no morphological differences between the different islands. Moreover the genetic distance between the specimens from Sumbawa and Flores is extremely low (Table 3), which either indicates ongoing gene flow between the islands or recent colonisation of one of the islands.

Müller-Liebenau (1984: p. 270–271, figs 14, 29, 47) described a Genus No.1 sp.1 from Malaysia, which most probably represents a new species of *Indocloeon*. However, it remains uncertain, if the stout, acute, bipectinate setae typical for *Indocloeon* are present on femur, tibia, and tarsus, as fig. 14h in Müller-Liebenau (1984) is not detailed enough and nothing is mentioned in the description. Genus No.1 sp.1 presents similarities to *I. indonesiae* and *I. timorense* sp. n., but also clear differences. It differs from *I. indonesiae* in the number of large apical denticles of the claw (two in Genus No.1 sp.1 and four in *I. indonesiae*), the left prostheca (fig. 14e in Müller-Liebenau 1984, fig. 16 in Kluge 2012) as well as in the shape of the postero-lateral extension of the paraproct (cercotractor) (fig. 14g in Müller-Liebenau 1984, fig. 8 in Kluge 2012). From *I. timorense* sp. n. it differs in the composition and arrangement of the submarginal arc of setae of the labrum, the maxillary palps (less slender in *I. timorense* sp. n.), the number of large apical denticles of the claw (two in Genus No.1 sp.1, five in *I. timorense* sp. n.) and the spines at the posterior margin of the abdominal terga (figs 14a, d, e, k, 47 in Müller-Liebenau 1984, Figs 4a, g, 5b, d in this study).

The presently known distribution of the genus *Indocloeon* encompasses distant areas like the Indian peninsula on one hand and some South Asian islands on the other hand. Similar distributions can be found in other lineages of Baetidae such as *Liebebiella* Waltz and McCafferty or *Chopralla* Waltz and McCafferty (Kluge et al. 2014, Marle et al. 2016). Because of the poor state of knowledge of most faunas in the Oriental realm (Gattolliat and Nieto 2009),we may expect more new species of *Indocloeon* to be discovered in this area through further collection efforts in the future, which may fill the gaps in its distribution range.

## Supplementary Material

XML Treatment for Indocloeon spathasetis

XML Treatment for Indocloeon timorense

## References

[B1] Baker K Chadwick M Sulaiman ZH (2016a) Eco-hydromorphic classification for understanding stream macroinvertebrate biodiversity in Brunei Darussalam, Northern Borneo. Zoological studies 55(37): 1–27. https://doi.org/10.6620/ZS.2016.55-37 10.6620/ZS.2016.55-37 PMC6511898 31966182

[B2] Baker K Chadwick M Kahar RS Sulaiman ZH Wahab RHA (2016b) Fluvial biotopes influence macroinvertebrate biodiversity in South-East Asian tropical streams. Ecosphere 7(12): 1–15. https://doi.org/10.1002/ecs2.1479

[B3] Baker K Chadwick M Wahab RAH Kahar RS (2017a) Benthic community structure and ecosystems functions in above- and below-waterfall pools in Borneo. Hydrobiologia 787(1): 1–16. https://doi.org/10.1007/s10750-016-2975-4

[B4] Baker K Chadwick M McGill RAR Wahab RHA Kahar RS (2017b) Macroinvertebrate trophic structure on waterfalls in Borneo. Marine and Freshwater Research. https://doi.org/10.1071/MF16373

[B5] Ball SL Hebert PDN Burian SK Webb JM (2005) Biological identifications of mayflies (Ephemeroptera) using DNA barcodes. Journal of the North American Benthological Society 24: 508–524. https://doi.org/10.1899/04-142.1

[B6] Dallwitz MJ (1980) A general system for coding taxonomic descriptions. Taxon 29: 41–46. https://doi.org/10.2307/1219595

[B7] Dallwitz MJ, Paine TA, Zurcher EJ (1999 onwards) User’s guide to the DELTA Editor. Version: 16 November 2016. http://www.delta-intkey.com

[B8] Folmer O Black M Hoeh W Lutz R Vrijenhoek R (1994) DNA primers for amplification of mitochondrial cytochrome c oxidase subunit I from divers metazoan invertebrates. Molecular Marine Biology and Biotechnology 3: 294–299. http://www.mbari.org/staff/vrijen/PDFS/Folmer_94MMBB.pdf 7881515

[B9] Gattolliat J-L Nieto C (2009) The family Baetidae (Insecta: Ephemeroptera): synthesis and future challenges. Aquatic Insects 31: 41–62. https://doi.org/10.1080/01650420902812214

[B10] Hebert PDN Cywinska A Ball SL DeWaard JR (2003) Biological identifications through DNA barcodes. Proceedings of The Royal Society B-Biological Sciences 270: 313–321. https://doi.org/10.1098/rspb.2002.2218 10.1098/rspb.2002.2218 PMC1691236 12614582

[B11] Kimura M (1980) A simple method for estimating evolutionary rates of base substitutions through comparative studies of nucleotide sequences. Journal of Molecular Evolution 16: 111–120. https://doi.org/10.1007/BF01731581 10.1007/BF01731581 7463489

[B12] Kluge NJ (2012) Non-African representatives of the plesiomorphion Protopatellata (Ephemeroptera: Baetidae). Russian Entomological Journal 20: 361–376.

[B13] Kluge NJ Sivaramakrishnan KG Selvakumar C Kubendran T (2014) Notes about Acentrella (Liebebiella) vera (Müller-Liebenau, 1982) (= *Pseudocloeon difficilum* Müller-Liebenau, 1982 syn. n. = *Platybaetis arunachalae* Selvakumar, Sundar, and Sivaramakrishnan, 2012 syn.n.) (Ephemeroptera: Baetidae). Aquatic Insects 35: 63–70. https://doi.org/10.1080/01650424.2014.980272

[B14] Kumar S Stecher G Tamura K (2016) MEGA 7: molecular evolutionary genetics analysis version 7.0 for bigger data sets. Molecular Biology and Evolution 33: 1870–1874. https://doi.org/10.1093/molbev/msw054 27004904 10.1093/molbev/msw054 PMC8210823

[B15] Marle P Salles FF Gattolliat J-L (2016) Two new species of *Bungona* Harker, 1957 (Ephemeroptera: Baetidae) from Borneo, Indonesia. Zootaxa 4088(2): 221–235. https://doi.org/10.11646/Zootaxa.4088.2.4 27394336 10.11646/zootaxa.4088.2.4

[B16] Müller-Liebenau I (1982) A new genus and species of Baetidae from Sri Lanka (Ceylon): *Indocloeon primum* gen. n., sp. n. (Insecta, Ephemeroptera). Aquatic Insects 4: 125–129. https://doi.org/10.1080/01650428209361096

[B17] Müller-Liebenau I (1984) New genera and species of the family Baetidae from West-Malaysia (River Gombak) (Insecta: Ephemeroptera). Spixiana 7: 253–284.

[B18] Sanger F Nicklen S Coulson AR (1977) DNA sequencing with chain-terminating inhibitors. Proceedings of the National Academy of Sciences U.S.A. 74: 5463–5467. https://doi.org/10.1073/pnas.74.12.5463 10.1073/pnas.74.12.5463 PMC431765 271968

[B19] Vuataz L Sartori M Wagner A Monaghan MT (2011) Toward a DNA taxonomy of Alpine *Rhithrogena* (Ephemeroptera: Heptageniidae) using a mixed Yule-Coalescent Analysis of mitochondrial and nuclear DNA. PLoS ONE 6: 1–11. https://doi.org/10.1371/journal.pone.0019728 10.1371/journal.pone.0019728 PMC3096624 21611178

[B20] Zhou X Jacobus LM DeWalt RE Adamowicz SJ Hebert PDN (2010) Ephemeroptera, Plecoptera, and Trichoptera fauna of Churchill (Manitoba, Canada): insights into biodiversity patterns from DNA barcoding. Journal of the North American Benthological Society 29: 814–837. https://doi.org/10.1899/09-121.1

